# Tool Wear Prediction When Machining with Self-Propelled Rotary Tools

**DOI:** 10.3390/ma15124059

**Published:** 2022-06-07

**Authors:** Usama Umer, Syed Hammad Mian, Muneer Khan Mohammed, Mustufa Haider Abidi, Khaja Moiduddin, Hossam Kishawy

**Affiliations:** 1 Advanced Manufacturing Institute, King Saud University, Riyadh 11421, Saudi Arabia; smien@ksu.edu.sa (S.H.M.); muneerkm@ksu.edu.sa (M.K.M.); mabidi@ksu.edu.sa (M.H.A.); khussain1@ksu.edu.sa (K.M.); 2 Machining Research Laboratory, University of Ontario Institute of Technology, Oshawa, ON L1G 0C5, Canada; hossam.kishawy@uoit.ca

**Keywords:** hard turning, self-propelled rotary tools, flank wear, genetic programming

## Abstract

The performance of a self-propelled rotary carbide tool when cutting hardened steel is evaluated in this study. Although various models for evaluating tool wear in traditional (fixed) tools have been introduced and deployed, there have been no efforts in the existing literature to predict the progression of tool wear while employing self-propelled rotary tools. The work-tool geometric relationship and the empirical function are used to build a flank wear model for self-propelled rotary cutting tools. Cutting experiments are conducted on AISI 4340 steel, which has a hardness of 54–56 HRC, at various cutting speeds and feeds. The rate of tool wear is measured at various intervals of time. The constant in the proposed model is obtained using genetic programming. When experimental and predicted flank wear are examined, the established model is found to be competent in estimating the rate of rotary tool flank wear progression.

## 1. Introduction

Demanding materials like hardened steel confront issues during machining owing to their poor heat conductivity, resulting in focalized high temperatures [1]. This issue has a substantial impact on tool life because it causes an elevated incidence of tool wear, which lowers machining quality and yield [2]. The chemical interaction of difficult-to-cut materials with tool materials culminates in tool failure that is sudden and unexpected, as well as a poor surface finish [3]. Various strategies were used in the previous research to diffuse the accumulated heat. Coolant (or lubricants) have, for example, been frequently utilized to disperse and lessen the influence of induced heat, allowing the cutting domain temperature to be maintained inside a tolerable range [4,5,6,7]. The oils and liquids applied in the machining region function as a lubricant, reducing the degree of engagement amongst the chip and the tool and forming a thin layer [8]. The benefits of machining with coolant/lubricant are evident, but the usage of a coolant/lubricant has major consequences for humans and the environment [9]. Researchers have explored machining materials without using cutting fluids, often known as dry machining, to avoid deleterious cutting fluids during machining processes [10]. The most prevalent machining processes for difficult-to-cut materials have been hard turning and grinding [11]. As reported in [12], grinding has poor throughput and restricted capabilities in terms of flexibility and machined geometries. Turning difficult-to-cut machine materials instead of grinding, on the other hand, produces a high-quality machined surface at a lower cost [13]. The adoption of turning instead of grinding and without cutting fluids for hardened steel and other hard-to-machine materials has attracted industrial interest in recent years [14].

Dry hard turning lowers processing time and specific cutting energy consumption, as well as the healthcare and ecological problems associated with typical coolant-based machining processes [15]. Hard turning, on the other hand, has been impeded by significant tool wear [16,17]. As a result, regulating tool wear and its impact on the consistency of the workpiece surface has been a substantial technical dilemma.

## 2. Literature Survey

Hard turning necessitates tool materials featuring adequate wear and temperature resilience due to the tremendous specific forces and temperatures in the narrow interface zone across the tool and the machining surface. Furthermore, as established in [18], the indentation hardness of the tool must be thrice that of the machining surface. Ceramics and cubic boron nitride (*cBN*) tools are typically considered for hard turning since tool wear and plastic deformation of the cutting edge degrade the quality and consistency of the machining surfaces [19,20,21]. Many researchers have investigated the chip removal and wear mechanisms of hard turning employing *cBN*, polycrystalline cubic boron nitride (*PcBN*), and ceramic cutting tools. Sobiyi, et al. [22] explored the deterioration of ceramic and *PcBN* cutting tools during machining AISI 440B stainless steel under various machining settings. The cutting speed seemed to have the greatest impact upon the flank wear rate in the experiments, and it surged as the cutting speed grew for two cutting tools. With mixed ceramic tools, flank wear increased as feed increased, but with *cBN* cutting tools, the converse was truly attributable to severe tool vibration. The *cBN* tool also had a greater metal removal rate because of its rigidity and tendency to sustain its toughness at greater cutting speeds and feed rates. The primary wear mode for ceramic cutting tools was abrasive wear, while the primary wear modes for *cBN* tools were adhesive wear and abrasive wear. At reduced machining rates, both tools generated long, continuous, serrated cutting chips, but when the cutting speed rose over 150 m/min, the cutting chips become serrated and segmented. When turning hardened steel beyond 50 HRC, there was a propensity for increased tool wear intensity and exacerbated abrasion and diffusion wear processes, as documented [23]. It has also been reported that raising the hardness of the workpiece to 50 HRC reduced surface roughness, but raising the hardness of the workpiece between 50 to 65 HRC enhanced surface roughness [24]. The result of expanding workpiece hardness was thermal softening, substantial material lateral flows around the feed markings, and a compressing action on the tool flank face and the workpiece surface [25]. Tang, et al. [26] found that when the workpiece’s hardness level increased, so did the cutting forces. Tang, et al. [27] also studied the wear profiles and mechanics of *PcBN* tools in dry hard machining of AISI D2 hardened steel at varied hardness grades (40–60 HRC). The findings revealed that the hardness of the workpiece had a significant impact on flank wear. The primary wear modes in the flank wear of *PcBN* tools comprised abrasive wear in instances of 40–55 HRC, plus abrasive and delamination wear in scenarios of 60 HRC because of a sudden rise in resistance at tool-workpiece junctions, whereas the crater was the predominant wear in the rake surface of *PcBN* tools. One of the most important aspects of dry machining is the selection of the right cutting tool and its material [28]. The use of traditional tools and materials raises the cutting temperature in the cutting zone, resulting in rapid tool wear, which compromises the dimensional precision, surface roughness, and tool life of the workpiece. Carbides, for example, are not typically utilized for hard turning; instead, they are employed at low speeds for regular turning operations to maximize tool life [29]. In the same way, when doing hard machining, a single cutting point tool with only one main cutting edge has drawbacks. It has the downside of a rapid rate of wear and a high temperature at the cutting tool’s tip, which causes the tool to break prematurely. During machining, the tool is constantly in physical contact with the work material, causing a rapid rise in tool temperature, which accelerates tool wear and causes thermal damage to the machined surface. Furthermore, high-temperature fluctuations often plastically distort the tooltip, resulting in poor cutting accuracy. In the case of a single-point cutting tool, the material removal rate (*MRR*) is generally quite low. The ability of rotary cutting tools, as well as the properties of advanced cutting tool materials such as *cBN*, *PcBN*, and ceramic, to keep a viable cutting edge at high temperatures, has rendered them the most preferred alternative for machining hardened materials.

Rotary tools provide a cost-effective option to the conundrum of severe heat accumulation as well as maintain good tool performance during machining challenging materials in dry settings [30]. For example, Kishawy, et al. suggested in [14,31], that rotary tools significantly extended tool life, reduced cutting temperature, and enhanced *MRR*. Ezugwu [32] corroborated that which was described in [14,31], and maintained that rotary tools provided even surfaces, especially in the event of difficult-to-cut materials. As a result of its superior performance for difficult-to-machine materials, rotary tools have attracted a lot of attention from the machining community.

**Figure 1:**
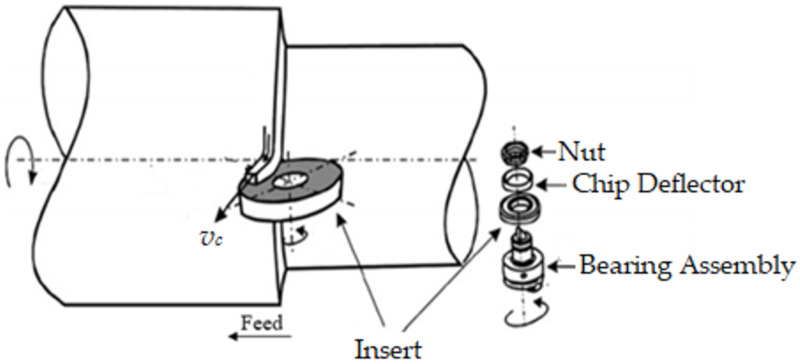
Characteristic rotary tool machining [14].

A rotary cutting tool is a disk-shaped cutting tool that revolves around its axis, as shown in Figure 1. In industry and research, two types of rotary tools are commonly used: driven and self-propelled. The insert’s rotating motion is generated by an independent source for the actively driven rotary tool (*ADRT*), whereas the chip progression across the tool rake surface forces the insert to revolve for self-propelled rotary tools (*SPRT*) [14]. Because the tool rotates, each segment of the cutting tip is involved in cutting over a minimal length of time, enabling every portion of the cutting surface to cool down after contact. When contrasted to standard tools, this results in intrinsically excellent cooling capabilities, allowing for the employment of cost-effective carbide inserts while hard turning. Exceptional wear tolerance and extended tool life have also been reported in earlier investigations, thus emphasizing rotary tools for hard machining. Ahmed, et al. [33] studied and evaluated the *SPRT*’s functionality in the dry machining of hardened steel alloy. The results confirmed that the *SPRT* provided smaller cutting forces, fairly low flank tool wear and extremely low machining temperature than conventional tools. When machining with *SPRT*, flank tool wear was curbed by 22 and 37% in the finest and harshest cutting circumstances, respectively. Similarly, when the cutting tool temperature was at its highest, it was discovered that the temperature was lowered by roughly 13%. Furthermore, when the tool temperature was at its lowest, the tool temperature was reduced by roughly 37%. As opposed to traditional tools, the use of *SPRT* can result in a significant decrease in power usage and a 20 times increase in tool life [34,35]. Dessoly, et al. [30] also noticed that a rotating tool had a 50 °C lower cutting temperature than a fixed tool. A hybrid model was demonstrated in [2] to precisely replicate and study the machining process using *SPRT*. An average cutting temperature drop of 65 °C was observed between the customary and rotating tools. In the case of the rotary tool, the highest temperature was found in the chip’s core and did not spread to the secondary shear zone. The maximal temperature in addition to the tool bulk temperature reduced as the tool rotational speed increased, but the temperature rose afterward above 900 rpm rotational speed. Umer, et al. [36] proposed a model for evaluating *SPRT* performance during hardened steel machining. A finite element (FE) model was conceived to analyze the hard turning of AISI 51200 and estimate cutting forces with greater precision and accuracy. The tool-chip engagement span for the rotary tool case was recorded as shorter because of the differences in chip flow angles. Temperatures were greater in the fixed tool, and the rotating tool’s surface temperature was assessed to be around 35% lower than that of the fixed tools. Kishawy and Wilcox [37] investigated the efficacy of rotary tools while hard turning AISI 4340 steel with a hardness of 54–56 HRC. In both fixed and rotating scenarios, the effectiveness of carbide and TiN coated carbide inserts were examined. When rotary tools were assessed against fixed tools at the identical cutting settings, there was no indication of crater wear, and they demonstrated significant tool flank wear endurance. Subsequently, Kishawy, et al. [31] investigated the efficacy of *SPRT* and the consistency of workpiece surfaces when processing waspaloy and titanium alloys. In rotary tools, equally disseminated flank wear was noticed to be the primary cause of tool collapse, whereas in regular non-rotary tools, crater wear was discovered to be the leading trend. The cutting edge’s rotating movement was responsible for this. Because the cutting edge is continuously replenished by rotation, thermally stimulated degradation was less of a concern than the insert’s morphological viability.

A variety of models have been presented to approximate the tool flank wear rate during machining. Li, et al. [38], for example, devised a theoretical model that encompasses both abrasive and adhesive wear in order to explore the mechanism of flank wear for tools constructed of various polycrystalline diamond (PCD) materials. The width of flank wear was estimated by computing the differential equation established to represent the rate of flank wear and its interaction with cutting parameters, tool characteristics, and the workpiece material. Choudhury, et al. [39] developed a tool wear model that quantitatively described the progression of tool wear in turning operations using parameters such as the index of diffusion, wear coefficient, and tool/workpiece hardness ratio. A prediction model was implemented in a work by Xiaoliang, et al. [40] to evaluate the depth of plastic deformation at various tool flank wear states. Bombiński, et al. [41] also designed a method for quickly diagnosing gradual tool wear (GTW) and catastrophic tool failure (CTF). This approach depended on analyzing the waveforms of the cutting force sensor signal in sequential time periods. Cutting forces increased when the flank wear area increased according to a study by Sikdar and Chen [42]. They constructed a mathematical model for a deeper insight into the correlation between flank wear region and cutting forces. Additionally, the mathematical model in [43] can be exploited to assess tool wear in a turning operation in real-time. The relation involving flank wear and the ratio of force components were derived for this purpose using data from several tests. FE models have also been used to investigate the wear mechanism in the machining of hard-to-cut materials in addition to analytical or mathematical models [44,45,46,47,48].

In the literature, *SPRT* has been demonstrated to be a very good alternative to hazardous cooling fluids and other techniques for minimizing extreme heat generation and preserving optimum tool operation. It does, in fact, offer a number of benefits, including superior cooling capabilities, less heat generation, increased tool life, improved *MRR*, lower cutting forces, higher wear tolerance, reduced power consumption, etc. When compared to *ADRT*, *SPRT* is a more cost-effective and feasible option for cutting difficult-to-machine materials. Although *ADRT* can provide more control, it requires an additional power supply, making it complicated and costly. A multitude of research has been published in the literature focusing on the wear mechanism for turning operations using typical single-point cutting tools. Likewise, a lot of analytical and FE models have been established to comprehend the wear mechanism in single-point cutting tools. However, there is a larger gap in the literature for understanding the tool wear mechanism of *SPRT* during hard machining, particularly when analyzing flank wear. As a result, the focus of this research is to establish a mathematical model that can be used to analyze the evolution of flank tool wear during the *SPRT* machining of hardened steel.

## 3. Tool Wear Model Development

**Figure 2:**
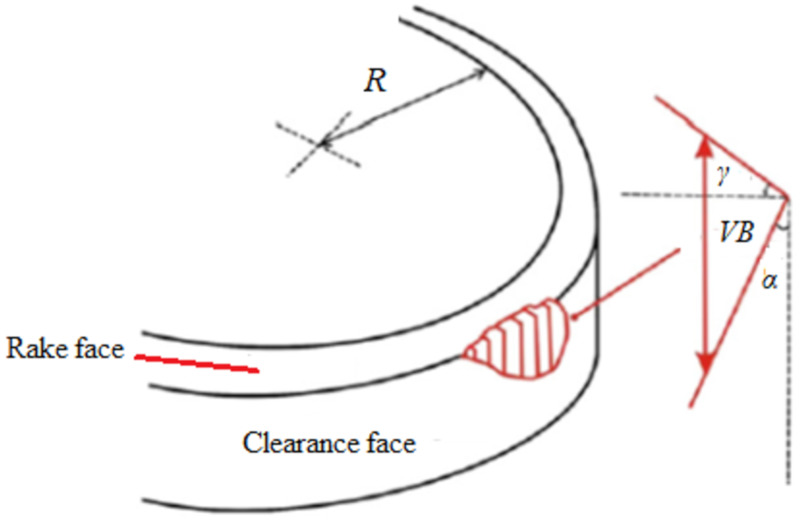
Cross-sectional interpretation of tool illustrating worn region based on the largest triangle [14].

The rubbing motion involving the cutting tool and the freshly produced workpiece surface causes flank wear to occur on the tool’s flank face. The flank wear is a worn land around the insert’s perimeter whenever the rotary tool is deployed. In this model, it is assumed that the rotary insert is fixed exactly like a single point tool to analyze the flank wear. Figure 2 depicts the schematic of the tool tip engaged with the workpiece.

The largest triangle, which defines the cross-section zone of the flank wear in the rotary tool, was analyzed to derive the flank wear area described in [14]. However, this assumption is not accurate, as the worn area in the flank face is irregular. In the modified model, a new variable called “*C*” is added to the equation of the flank wear area (*A*) to compensate/correct the irregularity effect (see Equation (1)), where *Ƴ* is the rake angle and *α* is the clearance angle. It is assumed that this new correction variable is linearly proportional to the maximum measured flank wear (*VB*) and can be computed using Equation (2).

(1) $$A=\frac{C\;\tan\alpha VB^{2\;}\;}{2\;(1+\tan\alpha \tan Ƴ)}$$

(2) $$C=\frac{2}{3}\;\left(VB/n\right)+1$$

where, *n* is a factor that is included to estimate the flank wear of the rotary tool. It is defined using Equation (3).

**Figure 3:**
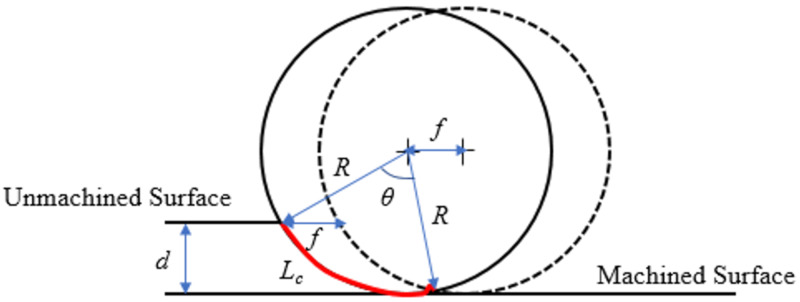
Cutting tool interacts with workpiece (top view) [14].

*n* = *L_c_*/2π*R*; *n* = /2π*R*; *n* = *θ/2π* (3)

where *L_c_* is the contact length between tool and workpiece as shown in Figure 3. When the depth of cut (*d*), and radius of the rotary tool (*R*) are known, Equation (4) could be used to compute the *L_c_*, which is the curve created by the angle *θ*. In this modified model, the second adaptation is done to simplify the contact length formula that was reported in [14]. In this work, the *L_c_* (Equation (4)) is expressed by excluding the feed rate term $\left(\tan^{-1}\left(f/2R\right)\right)$ because this term is relatively insignificant compared to the other term comprising *d* and *R*.

(4) $$L_{c}=R{\;\cos}^{-1\;}\left(\frac{R-d}{R}\right)$$

Figure 3 also depicts two circles representing the circumference of the rotary insert at two different workpiece rotations. Extruding the degraded region over the *L_c_* can be used to find the volume of the removed material (*V*). The rotary tool center traverses a linear distance equal to the feed after one revolution of the workpiece. The product of *A* and *L_c_* can be used to approximate the *V* on the flank land, as shown in Equation (5).

*V* = *L*_*c*_
*A* (5)

The volume lost due to abrasion and adhesion is linearly proportional to the cutting length *L*, as shown in Equation (6). The value of *L* is obtained by the product of cutting speed *v_c_* and the cutting time *t*.

*V* = *k*
*L* (*L* = *v*_*c*_
*t*) (6)

The third modification in the modified model is related to the empirical coefficient (*k*) concerning the interaction between the sliding interface pressure and the tool hardness. The coefficient *k* for a certain workpiece-tool pair can be described through the cutting time, feed rate, and cutting speed, as established by Dawson and Kurfess [49]. Thus, depending on the above modifications and using Equations (1)–(6), the formula for flank wear (*VB*) estimation can be expressed using Equation (7).

(7) $$\frac{2}{3}\;J{\left(\frac{VB}{n}\right)}^{3}+J\;{\left(\frac{VB}{n}\right)}^{2\;}-kv_{c}t=0$$

where *J* = $\frac{\cos^{-1\;}\left(\;\frac{R-d}{R}\right)\;R\;\tan\alpha }{2\;(1+\tan\alpha \;\tan Ƴ)}$ and *n* = $\frac{\cos^{-1\;}\left(\;\frac{R-d}{R}\right)\;}{2\;\pi }$.

Once the constant *k* has been established through a series of experimental trials, the flank wear model can be devised and used to estimate rotary tool flank wear at a given combination of cutting speed, feed rate, and cutting time.

## 4. Experimental Procedure

**Figure 4:**
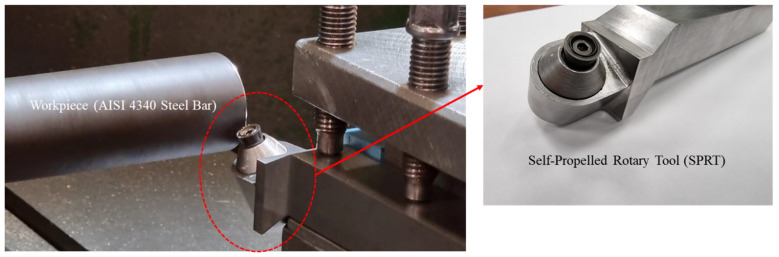
Experimental set up.

The tool wear model demonstrated above is verified by conducting longitudinal turning operations using *SPRT* on hardened steel bars (AISI 4340, 55 HRC) with 70 mm diameter and 200 mm length. The chemical composition of AISI 4340 steel is presented in Table 1 [50]. A number of variables that are important in the design of *SPRT* are considered in this investigation. For example, the *SPRT* must have a high inclination angle, be freely rotatable without interruption, and be built of a robust material capable of withstanding higher forces. The tool holder is designed in this study, while the round insert that is available on the market is chosen. The experiments are performed on an EMCO Concept TURN450 CNC lathe machine. Round coated carbide inserts with TiCN and Al_2_O_3_ coating is utilized with an in-house developed *SPRT* tool holder as shown in Figure 4.

The cutting tool geometric parameters are shown in Table 2. In order to find out the constant *k* for the tool wear model a total of six tool wear experiments are conducted with different cutting parameters.

After each cutting, test flank wear on the round insert is measured using the tool maker’s microscope. Table 3 depicts the cutting conditions and the tool flank wear at alternative cutting times. The depth of cut is set at 0.1 mm for all runs. The cutting conditions are chosen based on previous experience and preliminary trials, with the goal of achieving significant flank wear that can be accurately evaluated.

**Figure 5:**
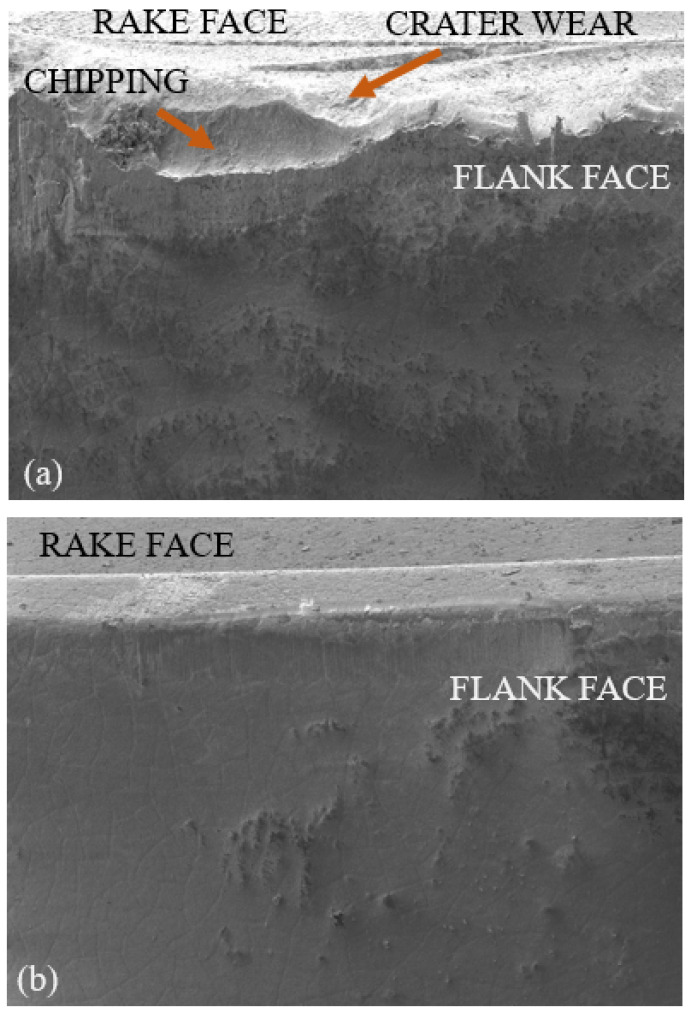
Flank faces of the worn cutting inserts (**a**) Fixed tool; (**b**) *SPRT*.

The same cutting inserts are also used with the fixed tool to have some comparative wear analysis with the *SPRT*. Figure 5 shows flank faces of the worn cutting inserts for fixed and rotary tools at a cutting speed of 100 m/min and a feed rate of 0.15 mm/rev after 3.5 min of cutting time. It can be seen that the coating is chipped-off for the fixed tool, whereas gradual flank wear can be observed for the rotating tool. It was also observed that for a standard tool life criterion of 0.3 mm flank wear, the rotating tool almost gives 12.5 times more cutting length as compared to the fixed tool. As depicted in Figure 5a, high edge chipping for the fixed tool leads to catastrophic failure most of the time in contrast to the rotating tool which shows gradual progressing of the flank wear until the end of tool life. In addition, little or no crater wear has been observed for the rotating tool owing to the self-cooling nature of the cutting edges as shown in Figure 5b.

The validity of the established tool wear model depends on the adequate estimation of the constant (*k*). The most often used approach for model identification is least squares regression (LSR). However, for nonlinear multivariable optimization problems, the iterative LSR is highly reliant on the assumed initial points due to its local sampling nature. The genetic programming (*GP*) based identification approach is, therefore, utilized to lessen the likelihood of becoming caught in the local optima [51,52]. Dawson and Kurfess [49] expressed *k* for a specific work-tool combination in terms of the cutting speed and the feed. In the current modified model, k is expressed in terms of cutting time, feed rate, and cutting speed using the *GP* modeling technique in MATLAB. *GP* is among the highly powerful artificial intelligence systems, and it is employed in a variety of engineering tasks. Every program in *GP* is made out of a tree structure of terminals and functions (i.e., genotype). The terminals (also known as leaves) are the network’s inputs, and the *GP* program’s functionalities comprise mathematical operations, programming capabilities, and arithmetic procedures. Every created model is represented as a chromosome, and each chromosome is evaluated using the fitness function. The fitness function calculates the difference between the output of the model and the training data input. The genetic operators, involving mutation and crossover parameters are used to make new chromosomes. Many pieces of research in the existing literature adopted *GP* to simulate their processes [53,54,55,56,57].

## 5. Results and Model Verification

The coefficient *k* for a definite work-tool pair is computed utilizing the data in Table 2 and Table 3. Table 4 illustrates the values of coefficient *k* that are derived empirically (*k_exp_*) using Equation (4). *GP* is then applied to build the prediction model premised on the experimental data.

**Figure 6:**
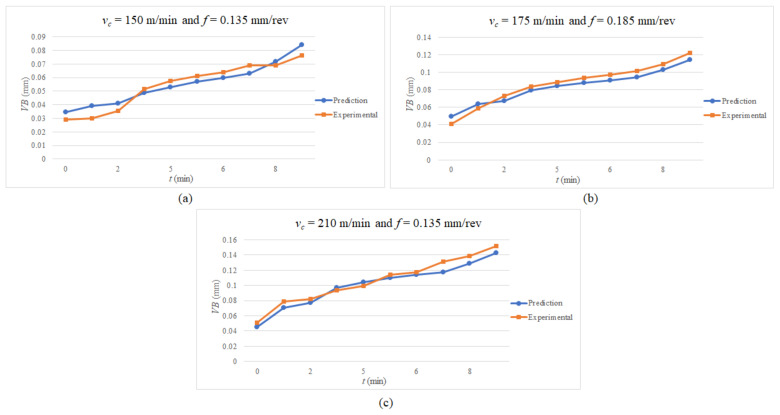
Flank wear at (**a**) *v_c_* = 150 m/min, *f* = 0.135 mm/rev; (**b**) *v_c_* = 175 m/min, *f* = 0.185 mm/rev; (**c**) *v_c_* = 210 m/min, *f* = 0.135 mm/rev.

The established flank wear model’s prediction scheme (from the *GP*) is subsequently exploited to corroborate the testing data by determining the value of “k” at various cutting speeds, feed rates, and cutting times. The test results are classified into three sets, each with its cutting speed, feed rate, and cutting time. The tool flank wear is monitored until it approaches 0.10 mm or 10 min of cutting time. The first set’s findings are acquired during machining at 150 m/min with a feed rate of 0.135 mm/rev, yielding an average accuracy of 89.35% (see Figure 6a). The mean accuracy for the second set (Figure 6b) is 92.23% while machining at a cutting speed of 175 m/min and a feed rate of 0.185 mm/rev. Lastly, the average accuracy of the final set, as seen in Figure 6c, is 92.71% during machining at a cutting speed of 210 m/min and a feed rate of 0.135 mm/rev. Thus, the model prediction and experimental observations have a high level of consistency. The established model is adept at estimating the coated carbide (with TiCN and Al_2_O_3_ coating) rotary tool flank wear while hard turning the AISI 4340 steel.

**Figure 7:**
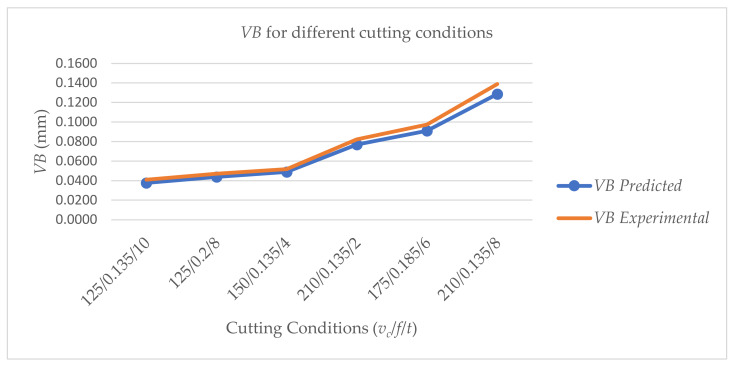
Prediction of flank wear randomly at various combinations of *v_c_*, *f*, and *t*.

The model is further confirmed by using a random combination of cutting speed, feed rate, and cutting time in addition to quantifying flank wear at various times. Again, the model performs well, with a 92.61% accuracy rate for forecasting flank wear, as shown in Figure 7.

## 6. Conclusions

A novel flank wear model for *SPRT* is established depending on the workpiece-tool geometric interactions and the empirical function. The new model assumes that the cross-section domain of the flank wear in the rotary tool is uneven (i.e., more realistic), whereas previous analyses considered it as regular (for simplicity). This new model also reduces the number of constants that must be estimated using some sort of optimization technique. As a result, reducing the number of constants in the new model improves its efficacy and accuracy. In this work, *GP* is used to estimate a constant in the model by hybridizing numerous cutting tests. It was discovered during cutting experiments and tool wear model building that the feed rate has a comparable consequence as the cutting speed on rotary tool flank wear evolution in hard turning for the speed range of 100–250 m/min. The suggested model’s usefulness is demonstrated by the satisfactory correlation among predicted and measured flank wear. The research is much more relevant for industry, especially when cutting hardened steel. The *SPRT* can assist industries in drastically lowering costs in mass production by minimizing tool wear and reducing environmental impact because no coolant is required. However, more experimental trials are needed to validate it, which is one of the goals of future research.

## Figures and Tables

**Table 1:** Chemical Composition of AISI 4340 Steel [50].

Element	C	Mn	P	S	Si	Ni	Cr	Mo
Composition (%)	0.38–0.43	0.60–0.80	0.035	0.040	0.15–0.35	1.65–2.00	0.70–0.90	0.20–0.30

**Table 2:** Cutting tool geometry parameters.

Rake angle (*γ*)	−5°
Clearance angle (*α*)	7°
Inclination angle (*λ*)	17°
Insert diameter (*D*)	16 mm
Cutting edge radius (*r*)	0.05 mm

**Table 3:** Cutting experiments for coefficient estimation.

Cutting Trials	Cutting Speed, *v*_*c*_ (m/min)	Feed, *f* (mm/rev)	Time, *t* (min)	Flank Wear, *VB*_*exp*_ (mm)
1	100	0.15	12	0.014
2	100	0.25	3.5	0.096
3	150	0.2	2	0.047
4	225	0.175	5.5	0.108
5	250	0.125	2.5	0.112
6	250	0.25	0.25	0.126

**Table 4:** Experimental and prediction values of coefficient *k*.

Run				Experimental	
	*v*_c_ (m/min)	*f* (mm/rev)	*t* (min)	*VB_exp_* (mm)	*k_exp_*
1	100	0.15	12	0.014	2.773 × 10^−5^
2	100	0.25	3.5	0.096	0.0115473
3	150	0.2	2	0.047	0.0020464
4	225	0.175	5.5	0.108	0.0045042
5	250	0.125	2.5	0.112	0.0098543
6	250	0.25	0.25	0.126	0.1363748

## Data Availability

The data presented in this study are available in the article.

## Abbreviations

*A*	Flank wear area
*C*	Constant
*cBN*	Cubic Boron Nitride
*D*	Insert diameter
*d*	Depth of cut
*f*	Feed
*GP*	Genetic programming
*k*	Empirical constant
*L_c_*	Contact length
*LSR*	Least squares regression
*MRR*	Material removal rate
*n*	Factor added to estimate the flank wear of the rotary tool
*PcBN*	Polycrystalline Cubic Boron Nitride
*R*	Radius of the rotary tool/insert
*r*	Cutting edge radius
*SPRT*	Self-propelled rotary tools
*t*	Cutting time
*VB*	Flank wear
*v_c_*	Cutting speed
*α*	Clearance angle
*γ*	Rake angle
*λ*	Inclination angle
